# Sexual violence against women by so-called Islamic state of Iraq and Syria (ISIS): protocol for a systematic review

**DOI:** 10.1186/s13643-020-01496-2

**Published:** 2020-10-12

**Authors:** Shadab Shahali, Shahrooz Shariati, Ali Montazeri

**Affiliations:** 1 Department of Reproductive Health and Midwifery, Faculty of Medical Sciences, Tarbiat Modares University, Tehran, Iran; 2 Department of Political Sciences, Faculty of Humanities, Tarbiat Modares University, Tehran, Iran; 3 Health Metrics Research Center, Institute for Health Sciences Research, ACECR, Tehran, Iran; 4 Faculty of Humanity Sciences, University of Science &Culture, ACECR, Tehran, Iran

**Keywords:** Sexual violence, Islamic state, Women, ISIS/IS, Systematic review

## Abstract

**Background:**

Violence against women and girls (VAWG) has been significantly increased by the rise of conflict and insecurity in the territories under controlling so-called Islamic State of Iraq and Syria (ISIS). This review aims to provide an understanding of the consequences of ISIS sexual violence against women.

**Methods:**

Electronic databases including MEDLINE, Cochrane Central Register of Controlled Trials, JSTOR, Web of Science, Scopus, Science Direct, ProQuest, and Google Scholar are searched for the articles published from 2014 to 2020. Then, two reviewers will systematically identify the articles which will meet the inclusion criteria. Using a standard checklist, methodological quality of articles is assessed. The findings will be summarized, and a narrative synthesis of data will be reported.

**Discussion:**

This systematic review with a narrative synthesis approach will provide the important information about the gap in knowledge and detailed summary of the existing evidence on consequences of ISIS's systematic sexual violence against women. The evidence is useful for the international health organizations to plan and develop clinical guidelines with interest to reduce the consequences of sexual violence in the armed conflict territories.

**Systematic review registration:**

PROSPERO CRD42019124215

## Background

The term sexual violence can include many different crimes ranging from sexual humiliation to multiple or gang rape and forced prostitution [1, 2]. It was defined with World Health Organization as “any sexual act attempts to obtain a sexual act, unwanted sexual comments or advances, or acts to traffic, or otherwise directed, against a person’s sexuality using coercion, by any person regardless of their relationship to the victim, in any setting, including but not limited to home and work” [3]. Sexual violence during wars and conflicts is not a new phenomenon [2]. It occurred all over the world which affected men and women for a long time [4]. However, until recently, it was a neglected issue [5]. Due to the global statistics, more civilian women were the target of violence in modern armed conflicts than civilian men for several reasons in the brutal way [1–3, 6–8].

Studies about conflicts usually staged the conflicts to “pre-conflict,” “the conflict itself,” “peace process,” and “reconstruction or reintegration” where at any stage, different consequences on victims are expected [9]. Women experience the conflict differently than men, so their challenges and needs are different during and after the conflict. The conflicts may have direct or indirect impacts on women. Direct impacts include sexual assault and rape, forced marriage, sexual slavery, disability, and the difficulty of accessing health care. Indirect impact includes honor killings, prostitution, rape, abduction, and trafficking [10].

Sexual violence, itself, has many physical and psychological consequences (e.g., homicide, suicide, serious injuries, pregnancy, sexually transmitted infections including HIV, suicide, and mental health problems), and it has negative impacts on society (e.g., influencing productivity and employment) [1].

The Middle East and North Africa region (MENA) faced the persistent violent conflicts and instability since late 2010 due to “Arab Spring.” When the conflict in Syria began in 2011, the Islamic State of Iraq (ISI) started the war in Syria, and ISIS which stands for “Islamic State of Iraq and Syria” was formed in April 2013 [11, 12].

The incident of violence against women and girls (VAWG) has been significantly increased with the rise of conflict and insecurity in the territories under controlling ISIS [13], especially among Yazidi, Christian, Turkomen, and Shabak women and girls aged between 8 and 35 years [11]. The United Nations Assistance Mission for Iraq (UNAMI) estimated that 1500 women and girls may be forced into sexual slavery. Studies showed that approximately 70% of women and girls who survived from ISIS captivity in Iraq were raped [13].

As stated, ISIS uses sexual violence widely, and few studies report on the sexual consequences of ISIS systematic sexual violence against women. ISIS is still a new phenomenon, and their use of sexual violence and its consequences have not been properly investigated. Thus, compiling evidence on the topic might help recognize the problem and perhaps provide the evidence for possible interventions. Currently, there is no systematic review addressing this phenomenon.

## Objectives

This review aims to provide an understanding of the consequences of ISIS’s sexual violence against women. We used the PICO tool (Table 1) to define the research question.

1. What forms does the violence take against women and young girls in ISIS territories?
2. What are the direct and indirect consequences of this violence on the victims?
3. What are the differences in the direct and indirect consequences of this violence on the victims who stayed in their home country and women and girls who became refugees?
4. What is the role of cultural and religious background with transgenerational trauma among Yazidi women and girls on the consequences of this new form of violence?

**Table 1:** The PICO framework to define the research question

**Population**	**Women and girls at the hands of ISIS in their territories**
**Intervention**	**Sexual violence**
**Comparators**	**Study not have a comparison group**
**Outcome**	**Direct and indirect consequences of sexual violence on the victims**

## Methods

### Information sources

Eight databases including MEDLINE, Cochrane Central Register of Controlled Trials, JSTOR, Web of Science, Scopus, Science Direct, ProQuest, and Google Scholar are searched for the articles published on the topic.

### Search strategy

**Fig. 1:**
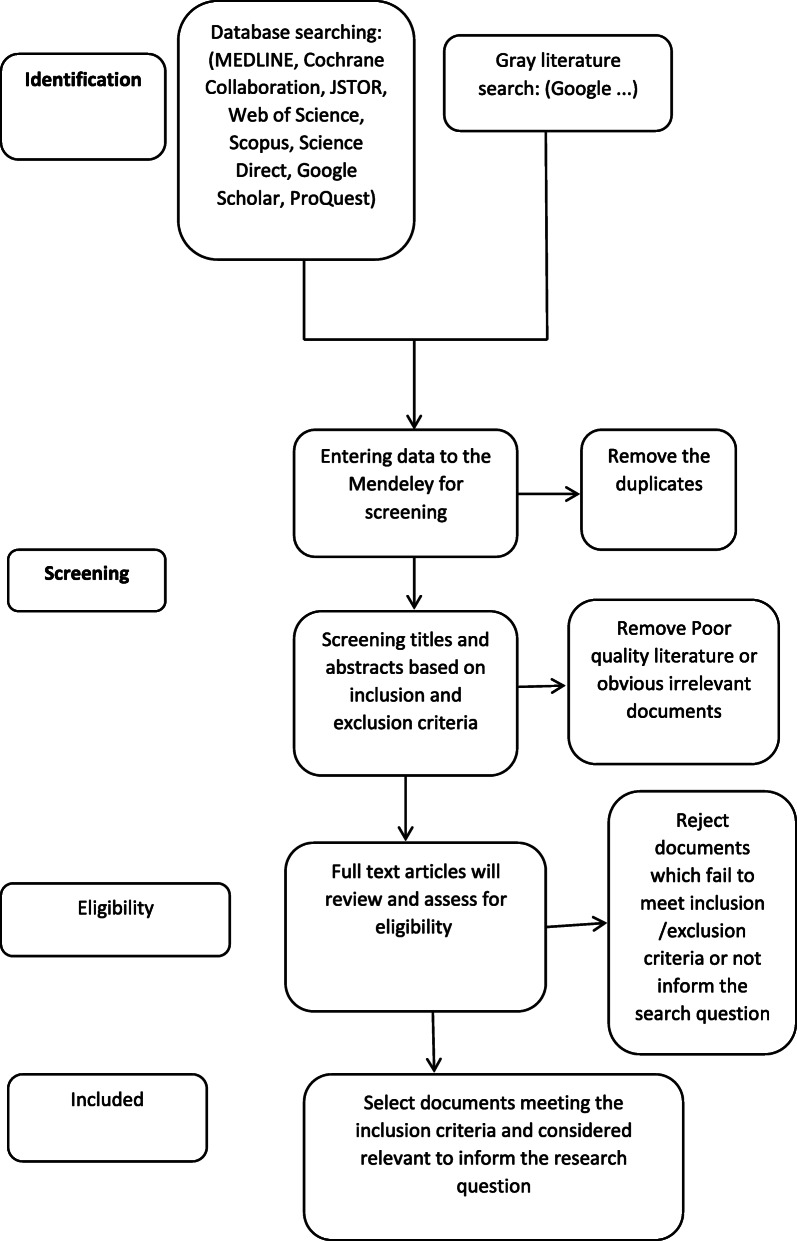
PRISMA (Preferred Reporting Items for Systematic Reviews and Meta-Analyses) flow chart review steps

Peer-reviewed journal articles and conference presentations with the qualitative or quantitative design will be sought. We will exclude the duplicates, narratives, expert opinions, and review articles. Our search terms will be the following: sexual violence OR sexualized violence OR captivity OR gender based violence OR rape OR religious violence OR terrorism OR Islamic terrorism OR political terrorism OR Islamic fundamentalism OR Jihad; AND IS OR ISIS OR ISIL OR Daesh OR Salafi OR Islamic State Or Jihadist Extremism OR Jihadi Terrorism; AND psychological consequences OR mental consequences OR cultural consequences OR economic consequences OR spiritual consequences OR physical consequences OR health related consequences OR trauma OR traumatized, AND women OR female OR gender OR girl. The sample MEDLINE search strategy is presented in Table 2. A PRISMA (Preferred Reporting Items for Systematic Reviews and Meta-Analyses) flow chart (Fig. 1) will present the review process’s steps. The search will be continued toward the end of study.

**Table 2:** Sample MEDLINE search strategy

1. MeSH descriptor: [Women] explode all trees.tw,ot.	
2. MeSH descriptor: [Female] this term only.tw,ot.	
3. MeSH descriptor: [Girl] this term only.tw,ot.	
4. Child.tw,ot.	
5. MeSH descriptor: [Adolescent] explode all trees.tw,ot.	
6. MeSH descriptor: [Young Adult] explode all trees.tw,ot.	
7. (OR/ 1-6) .tw,ot.	
8. MeSH descriptor: [Offense, Sex] explode all trees.tw,ot.	
9. MeSH descriptor: [Violence, Sexual] explode all trees.tw,ot.	
10. MeSH descriptor: [Abuse, Sexual] explode all trees.tw,ot.	
11. MeSH descriptor: [Violence, Gender-Based] this term only.tw,ot.	
12. MeSH descriptor: [Rape] this term only.tw,ot.	
13. MeSH descriptor: [Terrorism] this term only.tw,ot.	
14. MeSH descriptor: [Battered Women] explode all trees.tw,ot.	
15. MeSH descriptor: [Child Abuse] this term only.tw,ot.	
16. Coerc*.tw,ot.	
17. Captivity.tw,ot.	
18. Religious Violen*.tw,ot.	
19. Islamic terrorism.tw,ot.	
20. Political terrorism.tw,ot.	
21. Islamic fundamentalism.tw,ot.	
22. Jihad.tw,ot.	
23. Abuse near/3 women.tw,ot.	
24. Sex* near/3 abuse.tw,ot.	
25. Sex* near/3 coerc* .tw,ot.	
26. Sex* near/3 vioen*.tw,ot.	
27. Sex* near/3 assault.tw,ot.	
28. force* near/3 intercourse.tw,ot.	
29. (OR/ 8-28) .tw,ot.	
30. 7 AND 29.tw, ot.	
31. Islamic State OR IS.tw,ot.	
32. ISIS OR Islamic State in Iraq and Syria.tw,ot.	
33. ISIL OR Islamic State of Iraq and the Levant.tw,ot.	
34. Jihadist Extremism.tw,ot.	
35. Jihadi Terrorism.tw,ot.	
36. Daesh.tw,ot.	
37. Salafi.tw,ot.	
38. (OR/ 31-37) .tw,ot.	
39. 30 AND 38.tw,ot.	
40. Psychological Consequence*.tw,ot.	
41. Mental Consequence*.tw,ot.	
42. MeSH descriptor: [Psychiatric Disorder] explode all trees.tw,ot.	
43. Cultural Consequence*.tw,ot.	
44. Economic* consequence*.tw,ot.	
45. Spiritual consequence*.tw,ot.	
46. Physical consequence*.tw,ot.	
47. Health related consequence*.tw,ot.	
48. MeSH descriptor: [Reproductive Health] explode all trees.tw,ot.	
49. Trauma*.tw,ot.	
50. MeSH descriptor: [Psychological Trauma] explode all trees.tw,ot.	
51. MeSH descriptor: [Wounds and Injuries] explode all trees.tw,ot.	
52. Experience.tw,ot.	
53. Impression.tw,ot.	
54. Concern.tw,ot.	
55. Understand*.tw,ot.	
56. (OR/ 40-55) .tw,ot.	
57. 39 AND 56.tw,ot.	
58. MeSH descriptor: [Qualitative Research] this term only .tw,ot.	
59. MeSH descriptor: [Qualitative Analysis] this term only.tw,ot.	
60. MeSH descriptor: [Mixed Methods] this term only.tw,ot.	
61. MeSH descriptor: [Focus Groups] this term only.tw,ot.	
62. MeSH descriptor: [Narration] this term only.tw,ot.	
63. MeSH descriptor: [Interviews as Topic] explode all trees .tw,ot.	
64. MeSH descriptor: [Surveys and Questionnaires] this term only.tw,ot.	
65. MeSH descriptor: [Grounded Theory] this term only.tw,ot.	
66. Phenomenology.tw,ot.	
67. Ethnography.tw,ot.	
68. Content Analysis.tw,ot.	
69. Thematic Analysis.tw,ot.	
70. Discourse Analysis.tw,ot.	
71. Constant Comparative Analysis.tw,ot.	
72. Participant Observation.tw,ot.	
73. Narrative.tw,ot.	
74. Field Notes.tw,ot.	
75. (OR/58 - 74) .tw,ot.	
76. 7 AND 29 AND 38 AND 56 AND 75.tw,ot.

### Eligibility criteria

The present review will include all types of articles on sexual valence against women and girls which are published in English, German, French, and Arabic-languages from 2014 to 2020. The year 2014 was chosen, because ISIS was established. There will be no limitation by setting, and we will include all studies about sexual violence against women in ISIS territories.

### Study records

The results obtained from the literature search will be exported to Mendeley-Reference Management Software and Researcher Network. This Internet-based program simplifies the research team’s participation during the process of study selection. The research team will create a folder for the studies under review and develop subfolders to insert the databases’ results. The folder and subfolders will be shared with other research teams online to be assessed due to inclusion and exclusion criteria.

### Selection process

Two authors will screen the studies’ titles and abstracts using a designed screening tool, as shown in Table 3. After categorizing the studies, the full texts will be reviewed to finalize the categorization by employing independent double screening. Also, gray literature will be screened and categorized by the National Information Centre on Health Services Research and Health Care Technology at the National Library of Medicine (NICHSR) [14]. Finally, relevant documents will be examined and included.

**Table 3:** Preliminary articles screening tool

Criteria for selection	Yes	No	Cannot say
1. Publication date between 2014 and 2020			
2. English, German, French, or Arabic language			
3. Study participants are women and girls survivors’ of sexual violence			
4. Is related to sexual violence against women in the ISIS territories			

### Assessment of methodological quality and risk of bias individual studies

For quality assessment, Mixed Methods Appraisal Tool (MMAT) and scoring system will be used (Additional file 1) [15, 16]. The MMAT was adapted to check robustness of both qualitative and quantitative and for minimizing the risk of bias. In addition, if needed, we will contact the authors of papers for any possible issues which require clarification during data extraction and the double-check validation process.

### Data items

The data will be extracted as follows: psychological consequences, mental consequences, cultural consequences, economic consequences, spiritual consequences, physical consequences, and health-related consequences of ISIS’s systematic sexual violence against women, sample size, age, and type of violence, follow-up, outcome indicators, and the results.

### Outcomes

The outcome includes frequency and severity of sexual violence and its direct (physical) and indirect (psychological) consequences against women by ISIS. Physical or health-related consequences will include trauma, somatic problems, pregnancy, sexually transmitted infections, social isolation behavior, and sexual revictimization. Psychological or mental consequences will include the measures of suicidal thoughts or attempted suicide, depression, post-traumatic stress disorder (PTSD), stress, anxiety, sleep disorders, eating disorders, substance abuse, self-harm, panic attacks, quality of life, and self-esteem.

### Data synthesis (narrative synthesis)

We follow Popay et al.’s methodology for data synthesis, which involves four steps: “developing a theory of how the intervention works, why and for whom,” “developing a preliminary synthesis of findings,” “exploring relationships in data,” and “assessing the robustness of synthesis” [17].

1. Developing a theory of how the intervention works, why and whom for: Because of the exploratory nature of this review, the theory will not be developed.
2. Developing a preliminary synthesis of the findings of included studies: The studies’ descriptive characteristics will be tabulated. Then, the topics of interest will be checked to find out how the data can provide appropriate answers to the study questions.
3. Exploring relationships in the data. Exploring the relationships between and within studies will be carried out, and emerging strategies and characteristics patterns of studies will be rigorously evaluated.
4. Assessing the robustness of the synthesis: We will assess the strength of the evidence for concluding the consequences of sexual violence on the women and girls and assess our findings’ transferability to different contexts

## Discussion

This systematic review with a narrative synthesis approach will provide important information about the gap in knowledge and a detailed summary of the existing evidence on consequences of the ISIS’s systematic sexual violence against women. This evidence will help international health organizations plan and develop clinical guidelines with interest in reducing the consequences of sexual violence in the armed conflict territories. Our study’s strength is the use of validated and efficient tools for quality appraisal of reviews and study selection process. Our study’s limitations are that we will not use meta-analysis because studies are not reporting homogenous outcomes, and that too few studies exist to perform the statistical meta-analysis.

## Supplementary information

**Additional file 1:.** The Mixed Methods Appraisal Tool

## Data Availability

Not applicable.

## Abbreviations

IS: Islamic State
ISI: Islamic State of Iraq
ISIL: Islamic State of Iraq and the Levant
ISIS: Islamic State of Iraq and Syria
MENA: Middle East and North Africa
MMAT: Mixed Methods Appraisal Tool
NICHSR: National Information Center on Health Services Research and Health Care Technology
PRISMA: Preferred Reporting Items for Systematic Reviews and Meta-Analyses
PTSD: Post-traumatic stress disorder
VAWG: Violence against women and girls
